# Differential responses in placenta and fetal thymus at 12 days post infection elucidate mechanisms of viral level and fetal compromise following PRRSV2 infection

**DOI:** 10.1186/s12864-020-07154-0

**Published:** 2020-11-04

**Authors:** Angelica Van Goor, Alex Pasternak, Kristen Walker, Linjun Hong, Carolina Malgarin, Daniel J. MacPhee, John C. S. Harding, Joan K. Lunney

**Affiliations:** 1 Animal Parasitic Diseases Laboratory, Beltsville Agricultural Research Center, ARS, USDA, Beltsville, MD USA; 2 Department of Animal Sciences, Purdue University, West Lafayette, IN USA; 3 Department of Large Animal Clinical Sciences, Western College of Veterinary Medicine, University of Saskatchewan, Saskatoon, SK Canada; 4 College of Animal Science, South China Agricultural University, Guangzhou, China; 5 Department of Veterinary Biomedical Sciences, Western College of Veterinary Medicine, University of Saskatchewan, Saskatoon, SK Canada

**Keywords:** Porcine reproductive and respiratory syndrome, Gene expression, Fetal pig, Immunity, Placenta, Thymus, Cytokines, Interferon, Disease resistance, Disease susceptibility

## Abstract

**Background:**

A pregnant gilt infected with porcine reproductive and respiratory syndrome virus (PRRSV) can transmit the virus to her fetuses across the maternal-fetal-interface resulting in varying disease outcomes. However, the mechanisms leading to variation in fetal outcome in response to PRRSV infection are not fully understood. Our objective was to assess targeted immune-related gene expression patterns and pathways in the placenta and fetal thymus to elucidate the molecular mechanisms involved in the resistance/tolerance and susceptibility of fetuses to PRRSV2 infection. Fetuses were grouped by preservation status and PRRS viral load (VL): mock infected control (CTRL), no virus detected (UNINF), virus detected in the placenta only with viable (PLCO-VIA) or meconium-stained fetus (PLCO-MEC), low VL with viable (LVL-VIA) or meconium-stained fetus (LVL-MEC), and high VL with viable (HVL-VIA) or meconium-stained fetus (HVL-MEC).

**Results:**

The host immune response was initiated only in fetuses with detectable levels of PRRSV. No differentially expressed genes (DEG) in either the placenta or thymus were identified in UNINF, PLCO-VIA, and PLCO-MEC when compared to CTRL fetuses. Upon fetal infection, a set of core responsive IFN-inducible genes (*CXCL10*, *IFIH1*, *IFIT1*, *IFIT3*, *ISG15*, and *MX1*) were strongly upregulated in both tissues. Gene expression in the thymus is a better differentiator of fetal VL; the strong downregulation of several innate and adaptive immune pathways (e.g., B Cell Development) are indicative of HVL. Gene expression in the placenta may be a better differentiator of fetal demise than the thymus, based-on principle component analysis clustering, gene expression patterns, and dysregulation of the Apoptosis and Ubiquitination pathways.

**Conclusion:**

Our data supports the concept that fetal outcome in response to PRRSV2 infection is determined by fetal, and more significantly placental response, which is initiated only after fetal infection. This conceptual model represents a significant step forward in understanding the mechanisms underpinning fetal susceptibility to the virus.

**Supplementary information:**

**Supplementary information** accompanies this paper at 10.1186/s12864-020-07154-0.

## Background

Porcine reproductive and respiratory syndrome (PRRS) is a viral disease characterized by respiratory illness in growing pigs and reproductive failure in pregnant gilts [1]. The PRRS virus (PRRSV) is highly infectious, persistent, and variable. PRRS causes significant economic losses estimated at $664 million dollars annually in the U.S. alone [2]. Although biosecurity and vaccination have somewhat reduced losses from PRRS, more opportunities exist to incorporate favorable genetics and improve our understanding of host-pathogen interactions, especially in pregnant females.

Only during late gestation can PRRSV transplacentally infect the fetus resulting in mortality, still birth, and failure to thrive [3–5]. The pig has a non-invasive, epitheliochorial placenta where the maternal and fetal tissues are diffusely attached. The placenta protects the fetus while providing oxygen and nutrients from the mother to her fetuses, thus raising the question of the role it plays in transmission of PRRSV. Previous research has shown that PRRSV is detected in the endometrium and placenta causing apoptosis of infected and surrounding cells [6], but it is uncertain if apoptosis occurs before or after virus infects the fetus. Although the mechanism of transmission is not completely understood, there is an association with fetal infection and the number of sialoadhesion CD169 and CD163 positive macrophages (PRRSV permissible cells) present at the maternal fetal interface (MFI) [7, 8]. Trophoblast cells can be infected with PRRSV in vitro resulting in interruption of the cell cycle and can subsequently release viable virus capable of replication in other permissive cells [9]. Once virus infects the fetus, it can be found in various fetal tissues including thymus, lymph nodes, and blood. The thymus has been reported to be the major site of fetal viral replication and, thus, a major target for understanding the host response to infection [4].

Previous work found that fetuses at 21 days post infection (DPI) can be differentially impacted by PRRSV infection (e.g., viral load and disease outcome); fetuses are more likely to succumb to the infection if they have high viral loads and their neighboring fetuses are highly infected [10]. The cause of fetal demise is not well understood with debate on whether activities at the MFI (maternal endometrium and/or fetal placenta), activities in the fetus, or both are responsible, and to what degree [7]. Studies using gene expression have found common themes during fetal infection with PRRSV including changes in innate and adaptive immunity, hypoxia, interferon signaling, apoptosis, and thyroid hormone dysregulation [11–14]. Our current study is part of the largest set of studies to date using the pregnant gilt challenge model to understand phenotypic and genotypic responses to PRRSV infection in fetuses and gilts [15]. PRRSV was found to be translocated from dam to fetal placenta within 2 DPI, was first found in fetal thymus by 8 DPI, and 73% (36 of 49) of fetuses had detectable virus in their serum by 12 DPI [16]. Critical questions remain to understand transplacental PRRSV infection, especially the role of tissues at the MFI (e.g., placenta) and immune tissues in the fetus (e.g., the thymus).

This study used a pregnant gilt model with experimental PRRSV type 2 (PRRSV2) challenge at gestation day 85; fetal samples were collected at 12 DPI. The placenta, fetal thymus and serum were assayed for PRRS viral load (VL). Fetuses were grouped by preservation status as viable (VIA) or meconium stained (MEC). Additionally, fetuses were grouped by PRRS VL; uninfected (UNINF), virus in the placenta only (PLCO), low viral load (LVL), or high viral load (HVL). The fetal classifications described in the current study are based on previous work in our group [16]; UNINF, PLCO-VIA, and PLCO-MEC groups are resistant as they have, at least thus far, avoided or minimized infection suggesting some capacity to prevent viral entry/replication [17]. Fetuses classified as LVL-VIA and HVL-VIA are tolerant/resilient as they remain uncompromised in the face of viral infection [18], whereas the LVL-MEC and HVL-MEC are considered susceptible as they are neither able to limit viral replication and/or survive it [17]; fetuses with LVL have better outcomes than those with HVL. Our objective was to investigate targeted immune-related gene expression patterns and pathways in the placenta and fetal thymus to elucidate mechanisms associated with resistance/tolerance and susceptibility of fetuses to congenital PRRSV infection.

## Results

### Fetal groupings

**Fig. 1:**
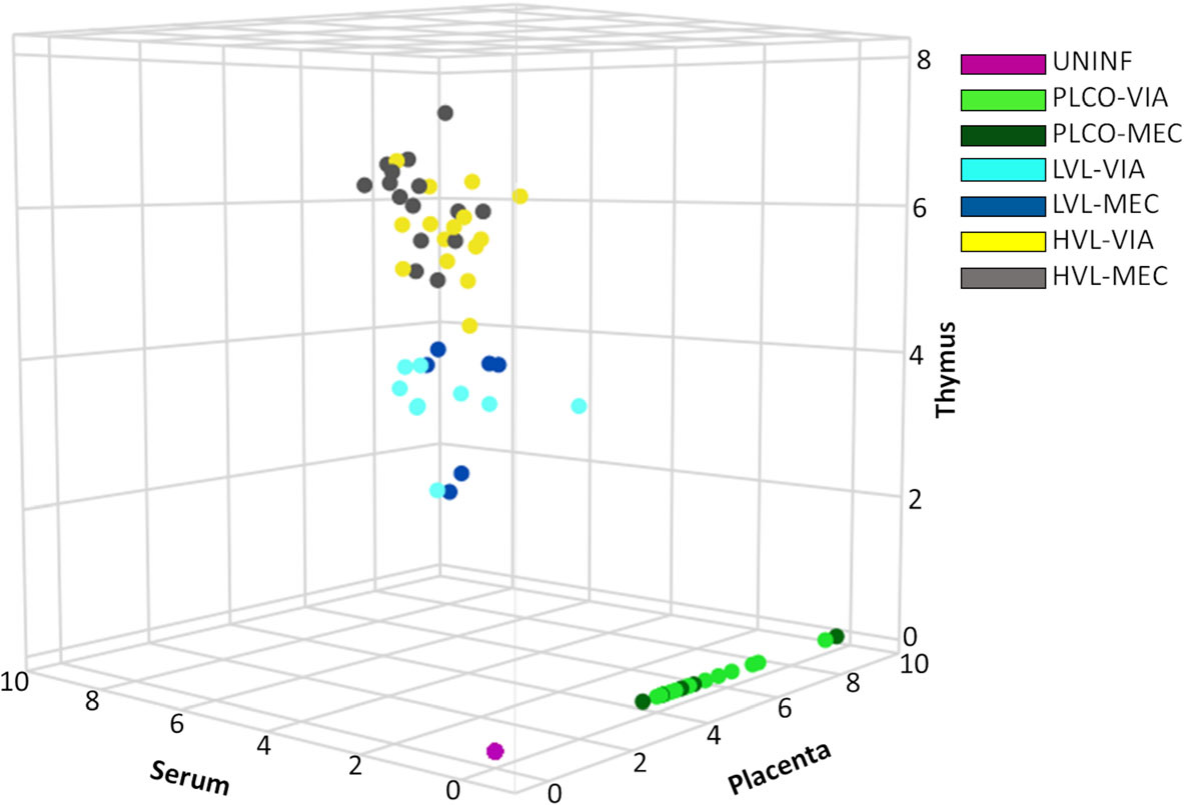
Fetal phenotypes for groups based on PRRSV viral load as determined by quantitative PCR on fetal placenta, serum, and thymus plotted in 3D with axes in log10 copies/μL as described previously [16]. Fetal groupings include uninfected (UNINF) as pink, placenta only with viable (PLCO-VIA) as light green, PLCO with meconium staining (PLCO-MEC) as dark green, low viral load with VIA (LVL-VIA) as light blue, LVL-MEC as dark blue, high viral load VIA (HVL-VIA) as yellow, and HVL-MEC as grey. Note that some samples are stacked (e.g., UNINF)

Our study attempted to probe the molecular mechanisms of fetal response to PRRSV infection. Our approach was to use the pregnant gilt model of infection to produce fetuses that varied in PRRS VL (UNINF, PLCO, LVL, and HVL) and preservation status (MEC and VIA). A 3D plot of the VL assessed in the fetal serum, placenta, and thymus used to determine fetal groupings is presented in Fig. 1. A full delineation of the fetal groupings can be found in Supplemental Figure 1. Detailed information pertaining to the numbers of fetuses in each group and their phenotypic characteristics including VL (fetal serum, placenta, and thymus) and morphometric measurements (fetal weight, crown rump length, and brain/liver ratio) is presented in Table 1. The fetuses in these groupings were characterized for differences in immune-related gene expression of the placenta and thymus (Table 2). Each fetal group was contrasted individually with CTRL; additionally, differentially expressed genes (DEGs) were calculated for larger biologically meaningful clusters of the previously described groups. In these larger contrasts, non-infected fetuses (UNINF, PLCO-VIA, and PLCO-MEC), referred to as V - F were used as the base to contrast with infected fetuses (LVL-VIA, LVL-MEC, HVL-VIA, and HVL-MEC), referred to as V + F group. Similarly, viable fetuses (UNINF, PLCO-VIA, LVL-VIA, and HVL-VIA), referred to as MEC - F, were used as the base to contrast with meconium stained fetuses (PLCO-MEC, LVL-MEC, and HVL-MEC), referred to as MEC + F group. We measured gene expression on the NanoString platform with a panel of 286 pre-selected genes (Supplemental Table 1) representing 22 pathways known or hypothesized to be impacted by PRRSV infection of either fetuses or nursery pigs.

**Table 1:** Summary of morphometrics and PRRSV viral load by fetal resilience groups

	Group	Classification	N	Placental viral load	Serum viral load	Thymus viral load	Fetal weight (g)	Crown rump length (cm)	Brain/ liver ratio
**Placenta**	**CTRL**	NA	15	ND	0 (0)	ND	757.7 (160.9)	27.2 (2.6)	1.4 (0.3)
	**UNINF**	R	16	0 (0)	0 (0)	0 (0)	753.2 (251.4)	26.8 (3.6)	1.3 (0.4)
	**PLCO-VIA**	R	12	5.3 (1.5)	0 (0)	0 (0)	751.5 (292.0)	26.8 (3.8)	1.5 (0.5)
	**PLCO-MEC**	R	4	5.6 (2.7)	0 (0)	0 (0)	868.2 (228.6)	28.8 (2.7)	1.3 (0.4)
	**LVL-VIA**	T	7	6.3 (1.45)	6.1 (1.1)	3.1 (0.5)	865.9 (200.1)	28.4 (3.1)	1.2 (0.3)
	**LVL-MEC**	S	6	7.4 (0.9)	7.1 (0.9)	3.1 (0.9)	798.0 (145.8)	27.4 (2.8)	1.4 (0.6)
	**HVL-VIA**	T	14	7.1 (1.1)	7.2 (0.8)	5.5 (0.6)	801.8 (257.8)	27.4 (2.8)	1.3 (0.4)
	**HVL-MEC**	S	12	7.3 (0.6)	8.2 (0.8)	6.1 (0.6)	732.0 (143.3)	25.5 (1.6)	0.8 (0.3)
**Thymus**	**CTRL**	NA	14	ND	0 (0)	ND	748.8 (163.9)	27.1 (2.7)	1.4 (0.3)
	**UNINF**	R	15	0 (0)	0 (0)	0 (0)	752.5 (260.2)	26.8 (3.7)	1.3 (0.5)
	**PLCO-VIA**	R	15	5.4 (1.5)	0 (0.01)	0 (0)	734.1 (274.6)	26.7 (3.6)	1.5 (0.4)
	**PLCO-MEC**	R	5	5.4 (2.4)	0 (0)	0 (0)	894.0 (206.2)	29.0 (2.4)	1.2 (0.4)
	**LVL-VIA**	T	8	6.3 (1.4)	6.4 (1.2)	3.1 (0.5)	839.6 (199.7)	28.1 (3.0)	1.3 (0.3)
	**LVL-MEC**	S	4	7.1 (1.0)	7.1 (1.1)	3.2 (0.9)	795.0 (173.4)	27.2 (2.8)	1.5 (0.8)
	**HVL-VIA**	T	15	7.2 (1.2)	7.2 (0.8)	5.6 (0.6)	789.1 (253.2)	27.2 (2.8)	1.4 (0.4)
	**HVL-MEC**	S	14	7.2 (0.6)	8.1 (0.8)	6.0 (0.7)	748.5 (127.3)	26.1 (1.7)	0.9 (0.3)

Fetal phenotypes for groups based on PRRSV viral load as determined by quantitative PCR on fetal placenta, serum and thymus, in the form of group average in log10 copies/μL. Numbers for phenotypic measurements are displayed as the mean and standard error in parentheses. Further subdivision of fetuses was made based on preservation status at the time of sample collection with only fetuses classified as either viable (VIA) and meconium stained (MEC) used in the present work. Group control (CTRL) was mock infected, gilt infected but no virus detected in the fetus (UNINF), virus detected in the placenta only with viable (PLCO-VIA) or meconium-stained fetus (PLCO-MEC), low viral load in the fetus with viable (LVL-VIA) or meconium-stained fetus (LVL-MEC), and high viral load in the fetus with viable (HVL-VIA) or meconium-stained fetus (HVL-MEC). Classifications; NA (not applicable as not challenged), resilient (R), tolerant (T), susceptible (S)

**Table 2:** Number and directionality of DEG by contrast

Contrast (group 1 – group 2)^a^		Placenta			Fetal thymus		
Group 1	Group 2	Down-Regulated DEG	Up-Regulated DEG	Total DEG	Down-Regulated DEG	Up-Regulated DEG	Total DEG
UNINF	CTRL	0	0	0	0	0	0
PLCO-VIA	CTRL	0	0	0	0	0	0
PLCO-MEC	CTRL	0	0	0	0	0	0
LVL-VIA	CTRL	0	11	11	1	8	9
LVL-MEC	CTRL	25	33	58	2	9	11
HVL-VIA	CTRL	36	46	82	84	30	114
HVL-MEC	CTRL	27	62	89	30	34	64
V + F (LVL-VIA + LVL-MEC + HVL-VIA + HVL-MEC)	V^−^ (UNINF + PLCO-VIA + PLCO-MEC)	77	47	124	97	35	132
MEC + F (PLCO-MEC + LVL-MEC + HVL-MEC)	MEC^−^ (UNINF + PLCO-VIA + LVL-VIA + HVL-VIA)	0	7	7	1	2	3

^a^Each group of fetuses was contrasted with the control (mock infected) group. Additionally, virus positive fetuses aka V + F (LVL-VIA + LVL-MEC + HVL-VIA + HVL-MEC) were contrasted with virus negative fetuses aka V - F (UNINF + PLCO-VIA + PLCO-MEC) as well as meconium-stained fetuses aka MEC + F (PLCO-MEC + LVL-MEC + HVL-MEC) versus viable fetuses aka MEC - F (UNINF + PLCO-VIA + LVL-VIA + HVL-VIA)

### Differential gene expression

The 286 test genes and 10 housekeeping genes investigated herein are detailed in Supplemental Table 1. The numbers and directions of DEG by contrast group and tissue are listed in Table 2. All DEG results from every contrast for every gene are listed in Supplemental Table 2. No DEG were identified in UNINF, PLCO-VIA, and PLCO-MEC, when each were contrasted to CTRL, within the corresponding tissue. In the LVL-VIA group, small numbers of DEG were identified with 11 in the placenta and 9 in the thymus; 95% (*n* = 19/20) of the total DEG were upregulated. In the LVL-MEC group, moderate numbers of DEG were identified in the placenta (*n* = 58) and 57% (*n* = 33/58) were upregulated, whereas the number of DEG identified in the thymus remained low (*n* = 11); 82% (*n* = 9/11) were upregulated. In the HVL-VIA group of fetuses, the number of DEG identified in the placenta remain moderate (*n* = 82), with 56% (*n* = 46/82) upregulated showing a similar expression pattern as LVL-MEC. By comparison, in the thymus of HVL-VIA fetuses we detected 114 DEG with 74% (*n* = 84/114) downregulated. In the HVL-MEC group 89 DEG were identified in the placenta, with 70% (*n* = 62/89) upregulated, and 64 DEG identified in the thymus, with 53% (*n* = 34/64) upregulated. The contrast of V + F had the highest numbers of DEG with 62% (*n* = 77/124) and 73% (*n* = 97/132) downregulated in the placenta and thymus, respectively. Very low numbers of DEG were identified in MEC + F group with 7 upregulated in the placenta, and 2 upregulated and 1 downregulated DEG in the thymus.

### Identification of unique and shared DEG

**Fig. 2:**
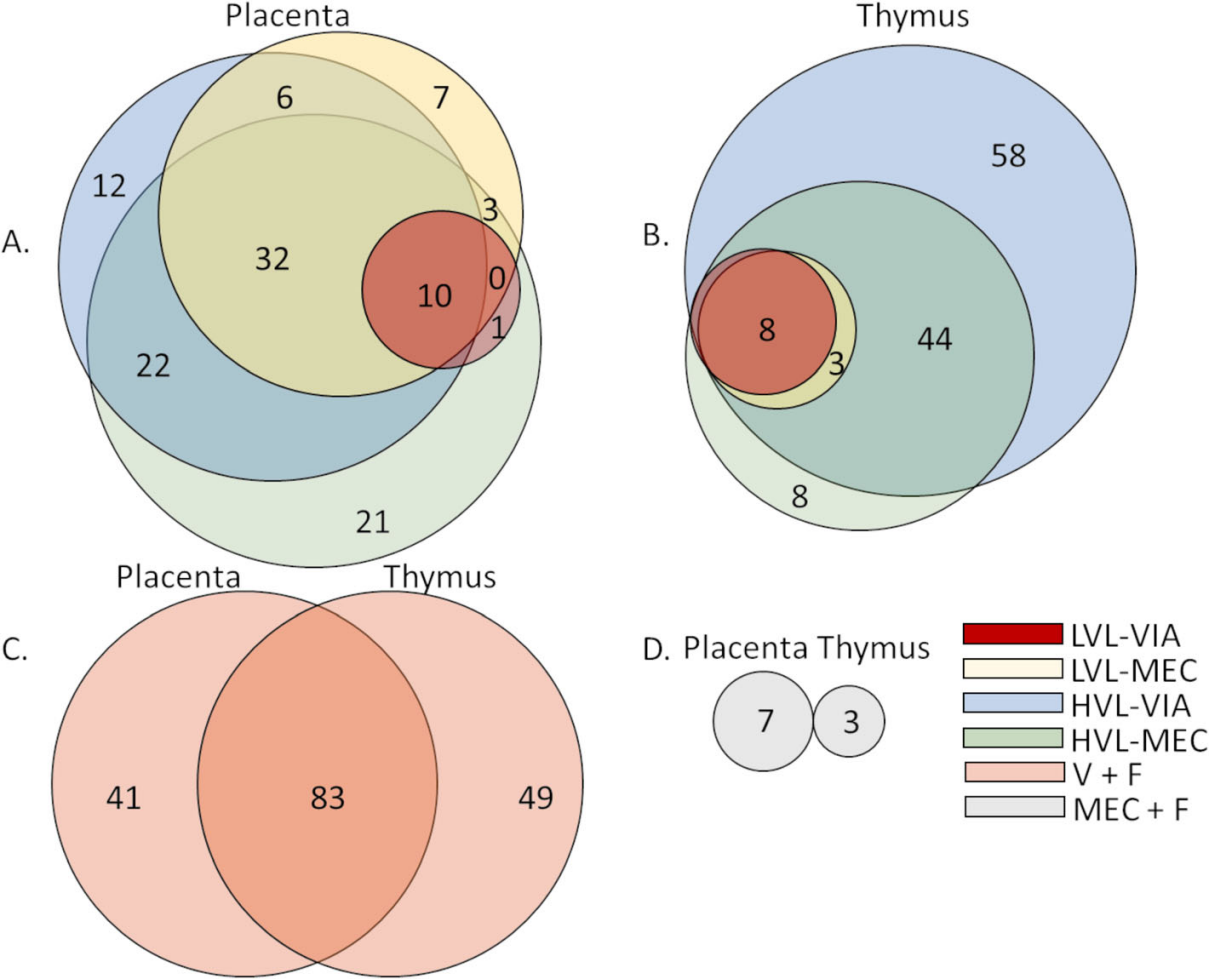
Unique and shared differentially expressed genes (DEG) in fetal tissues. Proportional-area Venn Diagrams: A) Placenta DEG each group contrasted to control (CTRL); low viral load with viable (LVL-VIA) or with meconium staining (LVL-MEC), high viral load with viable (HVL-VIA) or with meconium staining (HVL-MEC). B) Fetal thymus DEG each group contrasted to CTRL; LVL-VIA, LVL-MEC, HVL-VIA, and HVL-MEC. C) Placenta on the left and thymus on the right with DEG in V + F (LVL-VIA, LVL-MEC, HVL-VIA, and HVL-MEC) contrasted to V - F [UNINF, placenta only with VIA (PLCO-VIA), and PLCO-MEC]. D) Placenta on the left and thymus on the right with DEG in MEC + F (PLCO-MEC, LVL-MEC, and HVL-MEC) contrasted to MEC - F (UNINF, PLCO-VIA, LVL-VIA, and HVL-VIA). Bubble sizes are based on relative numbers of DEG for a given contrast calculated using the Euler method. Red is LVL-VIA, yellow is LVL-MEC, blue is HVL-VIA, green is HVL-MEC, orange is V + F, and grey is MEC + F^+^

**Fig. 3:**
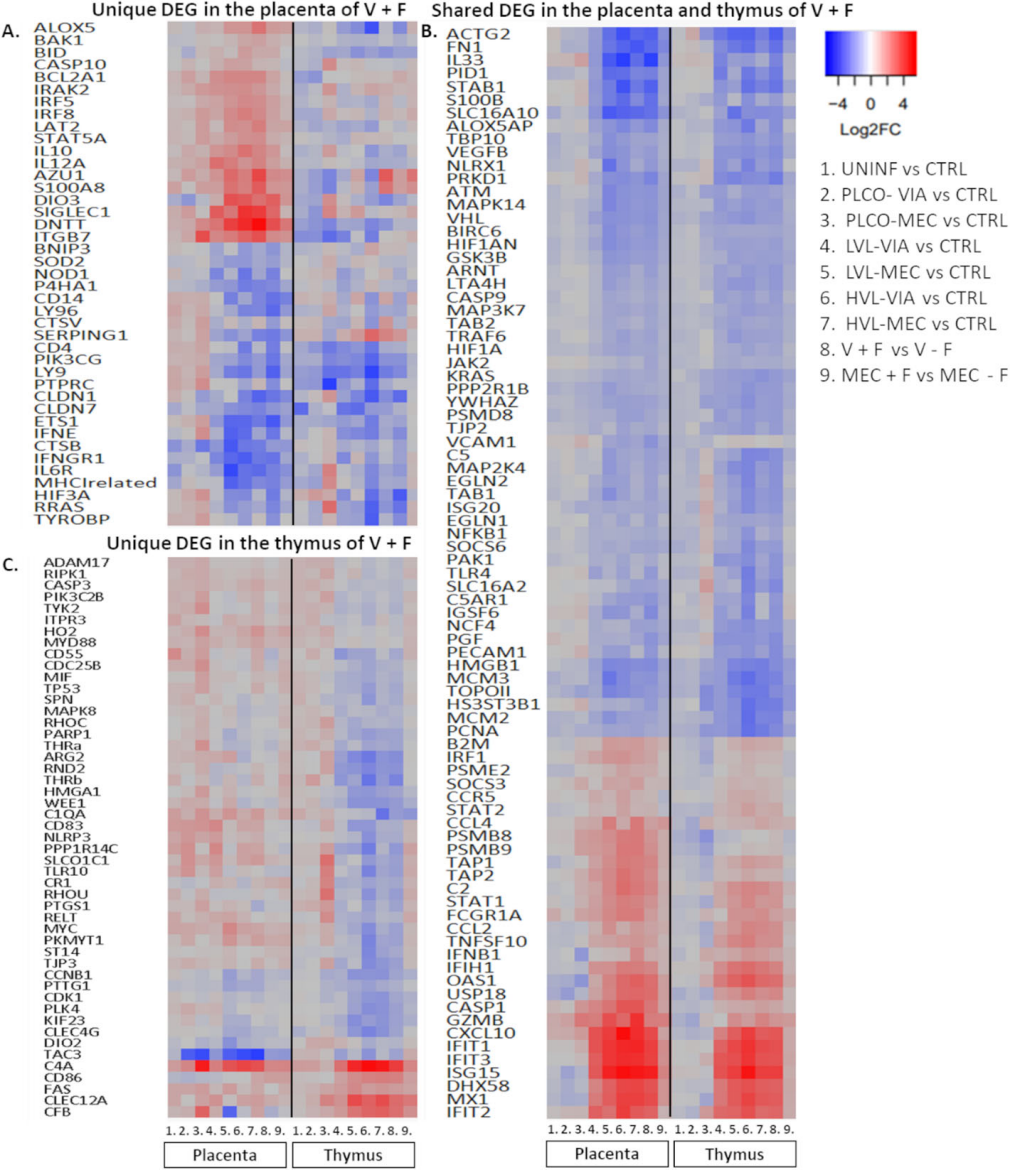
Heatmap of log2FC values with hierarchical clustering of genes based on V + F contrast uniquely differentially expressed genes (DEG) in the fetal placenta, thymus, or identified as DEG in both placenta and thymus. A) uniquely DEG genes in the placenta of V + F. B) DEG that were identified in both placenta and thymus of V + F. C) uniquely DEG in the thymus of V + F. The log2FC for each group was calculated in contrast to control (CTRL); 1. uninfected (UNINF), 2. placenta only with viable (PLCO-VIA), 3. PLCO with meconium staining (PLCO-MEC), 4. low viral load with VIA (LVL-VIA), 5. LVL-MEC, 6. high viral load VIA (HVL-VIA) and 7. HVL-MEC. 8. V + F calculated as (LVL-VIA, LVL-MEC, HVL-VIA, and HVL-MEC) contrasted to V - F (UNINF, PLCO-VIA, and PLCO-MEC). 9. MEC + F calculated as (PLCO-MEC, LVL-MEC, and HVL-MEC) contrasted to MEC - F (UNINF, PLCO-VIA, LVL-VIA, and HVL-VIA). The color corresponds to a gradient of the log2FC values ranging from downregulated in blue to upregulated in red

Unique and shared DEG were identified and visualized using proportional-area Venn Diagrams with overlap of individual groups contrasted with CTRL within tissue, as well as V + F and MEC + F groups’ overlap between tissues (Fig. 2). Within tissue comparison revealed a core of DEG in the PRRSV-infected (LVL-VIA, LVL-MEC, HVL-VIA, and HVL-MEC) fetal groups. There were 10 and 8 core DEG in the placenta and thymus, respectively. A large overlap (*n* = 6 of a possible 8) in the core DEG between the two tissues was identified and included *CXCL10*, *IFIH1*, *IFIT1*, *IFIT3*, *ISG15*, and *MX1*, all of which were upregulated (contrasted to CTRL). In the placenta, *CASP1*, *IFIT2*, *PSMB8*, and *STAT1* were the remaining tissue specific core DEG; in the thymus they were *DHX58* and *TNFSF10*. However, when we compared DEG between tissues within group (e.g., placenta HVL-MEC vs thymus HVL-MEC) we found only a moderate number of overlapping genes (Supplemental Figure 2), suggesting a unique immune-related gene expression response, above and beyond this core set contrast because this contrast had the largest number of DEG identified in both tissues. Uniquely DEG in the placenta and thymus, as well as the DEG identified in both tissues in the V + F contrast, are found in Fig. 3. Within the placenta V + F group, the top 5 unique DEG with the largest log2FCs were *DNTT*, *DIO3*, *SIGLEC1*, *AZU1*, and *ITGB7*, all of which were upregulated. Within the thymus V + F group, the top 5 unique DEG with the largest log2FCs were upregulated *C4A* and *CLEC12A*, and downregulated *THRb*, *ARG2*, and *RND2*. The top 5 shared DEG in placenta and thymus were *ISG15*, *IFIT1*, *IFIT3*, *CXCL10*, and *MX1* all of which were upregulated.

### Sample clustering based on fetal viral load and preservation status

**Fig. 4:**
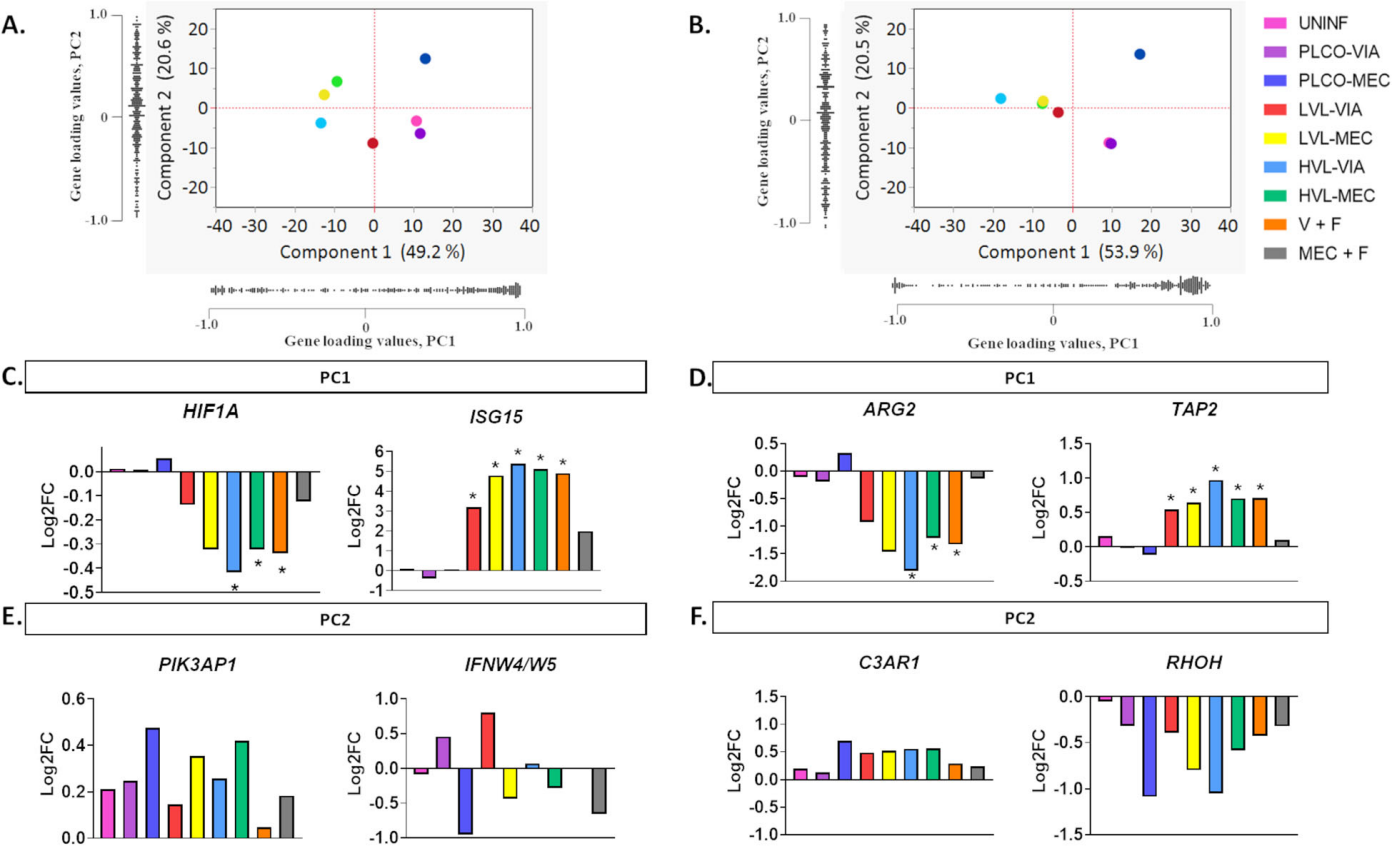
PCA score plots (A & B) showing placement of fetal preservation groups distinguished by viral load status (component 1) and viability (component 2). A) Placenta PCA plot. B) Thymus PCA plot. C) Placenta: the top positive and negative loaded genes on component 1 were *HIF1A* and *ISG15*, respectively*.* D) Thymus: the top positive and negative loaded genes on component 1 were *ARG2* and *C3AR1*, respectively*.* E) Placenta: the top positive and negative loaded genes onto component 2 were *PIK3AP1* and *IFNW4/W5*, respectively. F) Thymus: top positive and negative loaded genes for component 2 in the Thymus were *C3AR1* and *RHOH*, respectively. The log2FC for each group was calculated in contrasted to control (CTRL); uninfected (UNINF), placenta only with viable (PLCO-VIA), PLCO with meconium staining (PLCO-MEC), low viral load with VIA (LVL-VIA), LVL-MEC, high viral load VIA (HVL-VIA) and HVL-MEC. V + F calculated as (LVL-VIA, LVL-MEC, HVL-VIA, and HVL-MEC) contrasted to V - F (UNINF, PLCO-VIA, and PLCO-MEC). MEC + F calculated as (PLCO-MEC, LVL-MEC, and HVL-MEC) contrasted to MEC - F (UNINF, PLCO-VIA, LVL-VIA, and HVL-VIA). Pink is UNINF, purple is PLCO-VIA, dark blue is PLCO-MEC, red is LVL-VIA, yellow is LVL-MEC, blue is HVL-VIA, green is HVL-MEC, orange is V + F, and grey is MEC + F. *indicates significance *P* < 0.05 calculated in Limma

To investigate the relationship between fetal outcome groups based on our gene expression data we used principle component analysis (PCA) within tissues, using the log2FC expression of all 286 test genes calculated for each fetal group compared to CTRL. Additionally, the top two positively and negatively loaded genes for PCA components 1 and 2 for each tissue were plotted to look for informative expression patterns that could explain fetal VL and/or preservation status. The full list of gene loading values can be found in Supplemental Table 3. Results of the PCA with the log2FCs of the top positive and negative loaded genes for each tissue, are presented in Fig. 4. Principal component 1 explained 49 and 54% of the variance in the placenta (Fig. 4a) and thymus (Fig. 4b), respectively. In the score plots for both tissues, the fetuses grouped across component 1 based on their viral infection status i.e., viral negative fetuses were placed on the right side while viral positive fetuses were placed on the left side. Investigating the top positive and negative genes loaded onto component 1 revealed a strong and significant pattern of expression in both tissues. In the placenta, the top positive and negative loaded genes on component 1 were *HIF1A* and *ISG15* (Fig. 4c). In the thymus, the top positive and negative loaded genes on component 1 were *ARG2* and *TAP2* (Fig. 4d). In each tissue, component 2 captured approximately 21% of the variance (Fig. 4a & b). For placenta, fetuses grouped across component 2 based on their preservation status (i.e., with MEC scoring highly positive and viable fetuses highly negative) (Fig. 4a). The top positive and negative loaded genes onto component 2 in the placenta included *PIK3AP1* and *IFNW4/W5* (Fig. 4e). The fetal thymus samples separated somewhat for preservation status in component 2 (Fig. 4b) and the top positive and negative loaded genes for component 2 in the thymus were *C3AR1* and *RHOH*, respectively (Fig. 4f).

### DEG in the placenta for preservation status of fetuses

**Fig. 5:**
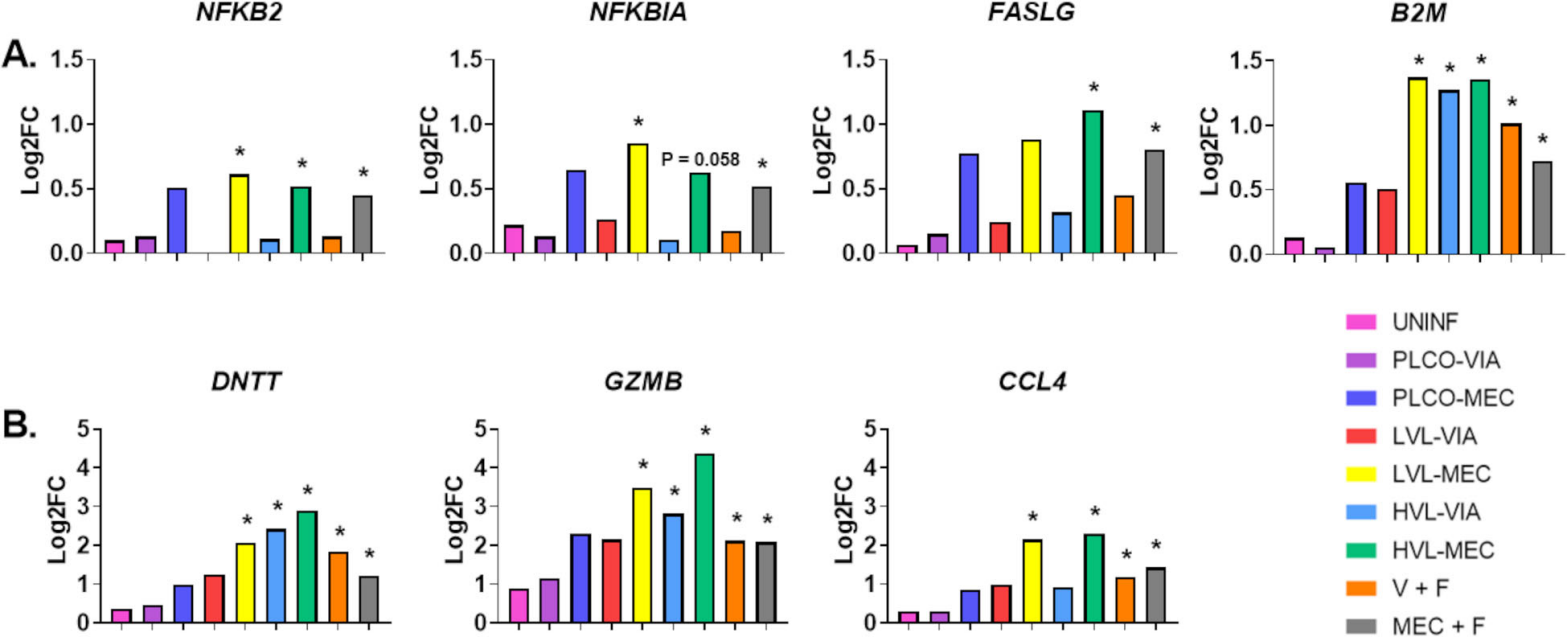
Biomarkers of fetal preservation in the placenta. Relative expression of 7 differentially expressed genes (DEG) found in the placenta in the MEC + F (PLCO-MEC + LVL-MEC + HVL-MEC vs UNINF + PLCO-VIA + LVL-VIA + HVL-VIA) contrast group; A) *NFKB2*, *NFKBIA*, *FASLG*, *B2M*, and B) *DNTT*, *GZMB*, and *CCL4* [Note difference in scale for A) versus B).]. The log2FC for each group was calculated in contrasted to control (CTRL); uninfected (UNINF), placenta only with viable (PLCO-VIA), PLCO with meconium staining (PLCO-MEC), low viral load with VIA (LVL-VIA), LVL-MEC, high viral load VIA (HVL-VIA) and HVL-MEC. V + F calculated as (LVL-VIA, LVL-MEC, HVL-VIA, and HVL-MEC) contrasted to V - F (UNINF, PLCO-VIA, and PLCO-MEC). MEC + F calculated as (PLCO-MEC, LVL-MEC, and HVL-MEC) contrasted to MEC - F (UNINF, PLCO-VIA, LVL-VIA, and HVL-VIA). Pink is UNINF, purple is PLCO-VIA, dark blue is PLCO-MEC, red is LVL-VIA, yellow is LVL-MEC, blue is HVL-VIA, green is HVL-MEC, orange is V + F, and grey is MEC + F. *indicates significance P < 0.05 calculated in Limma

The PCA analysis revealed fetal groups separated on component 2 for fetal preservation status in the placenta more so than the thymus. Therefore, we chose to further investigate patterns in the 7 DEG identified in the placenta in the MEC + F (vs MEC - F) contrast; *NFKB2*, *NFKBIA*, *FASLG*, *B2M*, *DNTT*, *GZMB*, and *CCL4*. Results are found in Fig. 5.

### Unique patterns of pathway enrichment revealed in fetal groups

We chose our NanoString gene set (*n* = 286) based on previously known or hypothesized PRRSV responsive genes and pathways (*n* = 22) with most genes annotated by Ingenuity Pathway Analysis (IPA) software and some manually assigned (Table 3). When virus was absent from the fetus (i.e., UNINF, PLCO-VIA, and PLCO-MEC) we did not detect any DEG in placenta or thymus; consequently, no pathway analysis was performed on data from these groups. In all other contrast groups, we performed a Core Analysis in IPA using DEG and their associated log2FC values as input. All data produced from the IPA Core Analyses are found in Supplemental Table 4.

**Table 3:** The investigated pathways verified by IPA

Pathway	Gene names^a^	Number of genes on NanoString assigned to pathway manually^b^	Number of genes on NanoString assigned to pathway by IPA^c^	Total number of genes in IPA pathway^d^	Coverage of IPA pathway (%)^e^
Acute Phase Response Signaling	*C2, C3, C4A, C4BPB, C5, CFB, FN1, FOS, IKBKB, IKBKE, IL1A, IL1RAP, IL33, IL6R, JAK2, KRAS, MAP2K4, MAP3K7, MAPK14, MAPK8, MYD88, NFKB1, NFKB2, NFKBIA, NFKBIE, OSM, PIK3CG, RIPK1, RRAS, SAA4, SERPING1, SOCS3, SOCS6, SOD2, STAT3, TAB1, TNF, TRAF2, TRAF6*	0	39	181	22%
Antigen Presentation Pathway	*B2M,* ***CTSS****, IFNG,* ***MHCI related****, PSMB8, PSMB9,* ***SIGLEC1****,* ***SLAMF6****, TAP1, TAP2*	4	6	39	15%
Apoptosis Signaling	*BAK1, BCL2A1, BCL2L1, BID, BIRC3, BIRC6,* ***BNIP3****, CASP10, CASP3, CASP8, CASP9, CDK1, FAS, FASLG,* ***GZMB****, IKBKB, IKBKE, KRAS, MAP2K4, MAPK8, MCL1,* ***MYC****, NFKB1, NFKB2, NFKBIA, NFKBIE, PARP1, PLCG1, PRKCQ, RRAS, TNF,* ***TOPOII****, TP53*	4	29	99	29%
B Cell Development	*CD19, CD40, CD79A, CD79B, CD86, DNTT, IL7R, PTPRC, SPN*	0	9	36	25%
B Cell Receptor Signaling	*BCL2A1, BCL2L1, BLNK, CD19, CD79A, CD79B, ETS1, FCGR2B, GSK3B, IKBKB, IKBKE, KRAS, LYN, MAP2K4, MAP3K7, MAPK14, MAPK8, NFKB1, NFKB2, NFKBIA, NFKBIE, PIK3AP1, PIK3C2B, PIK3CG, PRKCQ, PTK2B, PTPRC, RRAS,* ***TNFRSF13B***	1	28	190	15%
Complement System	*C1QA, C2, C3, C3AR1, C4A, C4BPB, C5, C5AR1, CD55, CFB, CR1, ITGAM, ITGB2, MASP1, SERPING1*	0	15	38	39%
HIF1-alpha Signaling	*ARNT, EGLN1, EGLN2, HIF1A,* ***HIF1AN****,* ***HIF3A****, KRAS, MAPK14, MAPK8, NOS3, PGF, PIK3C2B, PIK3CG, RRAS, TP53, VEGFA, VEGFB, VHL*	2	16	115	14%
HMGB1 Signaling	*CCL2, CXCL8, FASLG, FOS, HMGB1, ICAM1, IFNG, IFNGR1, IL12A, IL12B, IL1A, IL33, KRAS, MAP2K4, MAPK14, MAPK8, NFKB1, NFKB2, OSM, PIK3C2B, PIK3CG, RHOC, RHOH, RHOU, RND2, RRAS, TLR4, TNF, TNFSF10, VCAM1*	0	30	165	18%
IL-10 Signaling	*ARG2, CCR5, CD14, FCGR2B, FOS, IKBKB, IKBKE, IL10, IL1A, IL1RAP, IL33, MAP2K4, MAP3K7, MAPK14, MAPK8,* ***MIF****, NFKB1, NFKB2, NFKBIA, NFKBIE, SOCS3, STAT3, TAB1, TNF, TRAF6, TYK2*	1	25	73	34%
iNOS Signaling	*CD14, FOS, HMGA1, IFNG, IFNGR1, IKBKB, IKBKE, IRAK2, IRF1, JAK2, JAK3, LY96, MAPK14, MYD88, NFKB1, NFKB2, NFKBIA, NFKBIE, STAT1, TAB1, TLR4, TRAF6, TYK2*	0	23	48	48%
Interferon Signaling	*BAK1,* ***FCGR1A****,* ***IFIH1****, IFIT1,* ***IFIT2****, IFIT3, IFITM1, IFNA1/IFNA13, IFNB1,* ***IFND1/IFND2****,* ***IFND3/IFND4/IFND10****,* ***IFND5/IFND6/IFND9/IFND11****,* ***IFND7****,* ***IFND8****,* ***IFNE****, IFNG, IFNGR1,* ***IFNW2****,* ***IFNW4/W5****, IRF1, ISG15,* ***ISG20****, JAK2, MX1, OAS1, PSMB8, STAT1, STAT2, TAP1, TYK2*	12	18	36	50%
NFKB Signaling	*CASP8, CD40, FCER1G, GSK3B, IKBKB, IL1A, IL33, KRAS, LCK, MAP3K7, MAPK8, MYD88, NFKB1, NFKB2, NFKBIA, NFKBIE, PIK3C2B, PIK3CG, PRKCQ, PRKCZ, RIPK1, RRAS, TAB1, TAB2, TLR10, TLR2, TLR4, TLR7, TLR8, TLR9, TNF, TNFAIP3, TRAF2, TRAF6, ZAP70*	0	35	179	20%
Production of Nitric Oxide and Reactive Oxygen Species in Macrophages	*ARG2, FOS,* ***GP91-PHOX****, IFNG, IFNGR1, IKBKB, IKBKE, IRF1, IRF8, JAK2, JAK3, LYZ, MAP2K4, MAP3K7, MAPK14, MAPK8, MPO, NCF1, NCF2, NCF4,* ***NCR1****,* ***NCR2****, NFKB1, NFKB2, NFKBIA, NFKBIE, PIK3C2B, PIK3CG, PLCG1, PPM1J, PPP1R14C, PPP2R1B, PRKCQ, PRKCZ, PRKD1, RHOC, RHOH, RHOU, RND2, S100A8, SAA4,* ***SEPX1****, STAT1, TLR2, TLR4, TNF, TYK2*	4	43	194	22%
Protein Ubiquitination Pathway	*B2M, BIRC3, BIRC6, IFNG, PSMB8, PSMB9, PSMD8, PSME2, TAP1, TAP2, TRAF6, USP18, VHL*	0	13	277	5%
Senescence Pathway	*ATM,* ***AXL****, CCNB1, CDC25B, CDK1, CXCL8, ETS1, IFNA1/IFNA13, IFNB1, IFNK, IFNW1, IKBKB, IKBKE, IL1A, ITPR3,* ***KIF23****, KRAS, MAP2K4, MAP3K7, MAPK14,* ***MCM2****,* ***MCM3****,* ***MYC****, NFKB1, NFKB2, PARP1,* ***PCNA****, PIK3C2B, PIK3CG,* ***PKMYT1****,* ***PLK4****, PPM1J, PPP2R1B, PRKCZ,* ***PTTG1****, RRAS, SAA4, SOD2, TLR2, TP53, TRAF6, VHL,* ***WEE1***	10	33	280	12%
T Cell Receptor Signaling	***CD244****, CD3D, CD3E, CD3G, CD4,* ***CD48****, CD8A, CD8B, FOS, GRAP2, IKBKB, IKBKE, ITK, KRAS, LAT, LCK, LCP2,* ***LY9****, MAP2K4, MAPK8, NFKB1, NFKB2, NFKBIA, PIK3C2B, PIK3CG, PLCG1, PRKCQ, PTPRC, RRAS, TEC, ZAP70*	3	28	110	25%
Th1 and Th2 Activation Pathway	***CCL4****,* ***CCL8****, CCR5, CD3D, CD3E, CD3G, CD4, CD40, CD86, CD8A,* ***CLEC4G****, ICAM1,* ***ICAM3****, IFNA1/IFNA13, IFNG, IFNGR1, IL10, IL12A, IL12B,* ***IL1B1****, IL33, IL6R, IRF1, ITGB2, JAK2, JAK3, KLRD1,* ***LTA4H****,* ***LTC4S****, NFKB1, PIK3C2B, PIK3CG, PRKCQ, SOCS3, STAT1, STAT3, STAT5A, TYK2*	7	31	172	18%
Tight Junction Signaling	*ACTG2, CLDN1, CLDN3, CLDN4, CLDN7, FOS,* ***HS3ST3B1****, MYL4, MYL7, NFKB1, NFKB2, PPM1J, PPP2R1B, PRKCZ, TJP2, TJP3, TNF*	1	16	168	10%
Toll-like Receptor Signaling	*CD14, FOS, IKBKB, IL12A, IL12B, IL1A, IL33, IRAK2,* ***IRF5****, LY96, MAP2K4, MAP3K7, MAPK14, MAPK8, MYD88, NFKB1, NFKB2, NFKBIA, TAB1, TAB2, TLR10, TLR2, TLR4, TLR7, TLR8, TLR9, TNF, TNFAIP3, TRAF6*	1	28	77	36%
TR/RXR Activation	*DIO1, DIO2, DIO3, HIF1A, PIK3C2B, PIK3CG,* ***SLC16A10****, SLC16A2,* ***SLCO1C1****,* ***TG****,* ***THRa****,* ***THRb***	5	7	91	8%
TREM1 Signaling	*CASP1, CCL2, CD40, CD83, CD86, CXCL8, FCGR2B, ICAM1, IL10, ITGA5, JAK2, LAT2, MPO, MYD88, NFKB1, NFKB2, NLRP3, NOD1, PLCG1, STAT3, STAT5A, TLR10, TLR2, TLR4, TLR7, TLR8, TLR9, TNF, TREM1, TYROBP*	0	30	76	39%
Other^f^	*ADAM17, ALOX5, ALOX5AP, AZU1, CDH16, CLEC12A, CLEC7A, CTSB, CTSC, CTSV, CXCL10, CXCR2, DEFB1, DHX58, GBP5, HO2, IGSF6, ITGB7, MBL1, MDGA2, NLRX1, NPPA, P4HA1, PAK1, PECAM1, PID1, PTGS1, PTGS2, RELT, S100B, ST14, STAB1, TBP10, TTC7A, YWHAZ*	35	0	N/A	N/A
House Keeping^g^	*HMBS, HPRT1, IPO8, MAU2, RPL32, RPL4, SDHA, STX5, GAPDH, TOP2B*	10	0	N/A	N/A

^a^The gene names of those tested on the NanoString that were assigned to a given pathway by Ingenuity Pathway Analysis (IPA) software (accessed on February 27, 2020) with genes in bold manually assigned to the pathway based on relevant functional annotation from IPA

^b^The number of genes tested on the NanoString that were manually assigned to a pathway based on relevant functional annotation from IPA

^c^The number of genes tested on the NanoString that were assigned to a pathway by IPA software

^d^The total number of genes in the IPA database assigned to a given pathway

^e^The percent coverage of a given pathway calculated by dividing the columns (c/d)*100%

^f^Genes tested on the NanoString that were not assigned (automatically by IPA or manually) to any of the investigated pathways

^g^The genes tested on the NanoString used for normalization of the count data

**Fig. 6:**
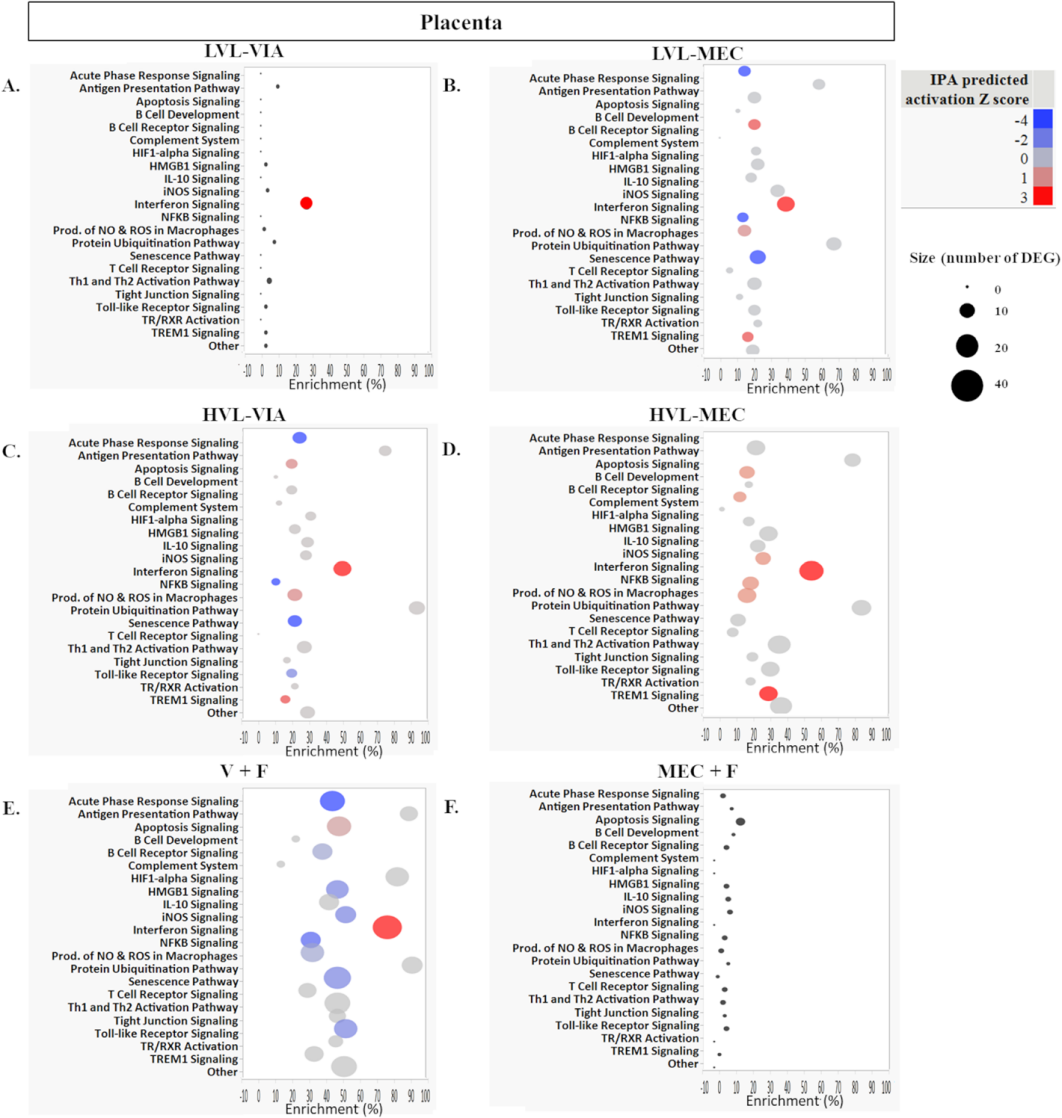
Pathway enrichment of differentially expressed genes in the placenta. A) low viral load with viable (LVL-VIA) contrasted to control (CTRL) fetuses. B) LVL with meconium staining (LVL-MEC) contrasted to CTRL fetuses. C) high viral load with viable (HVL-VIA) contrasted to CTRL fetuses. D) HVL with meconium staining (HVL-MEC) contrasted to CTRL fetuses. E) Virus positive fetus aka V + F (LVL-VIA, LVL-MEC, HVL-VIA, and HVL-MEC) contrasted to virus negative fetus V - F [UNINF, placenta only with VIA (PLCO-VIA), and PLCO-MEC]. F) Meconium stained fetus aka MEC + F (PLCO-MEC, LVL-MEC, and HVL-MEC) contrasted to viable fetus aka MEC - F (UNINF, PLCO-VIA, LVL-VIA, and HVL-VIA). The percent enrichment was calculated as the [(number of DEG assigned to a given pathway by IPA + the number of DEG manually assigned)/(total number of genes assayed on the NanoString in the given pathway)*100]. The pathway analysis plotted with pathway name on the y axis, enrichment (%) on the x, bubble size as the total number of DEG in each pathway, and the color as the IPA predicted activation Z score with blue deactivated and red activated

**Fig. 7:**
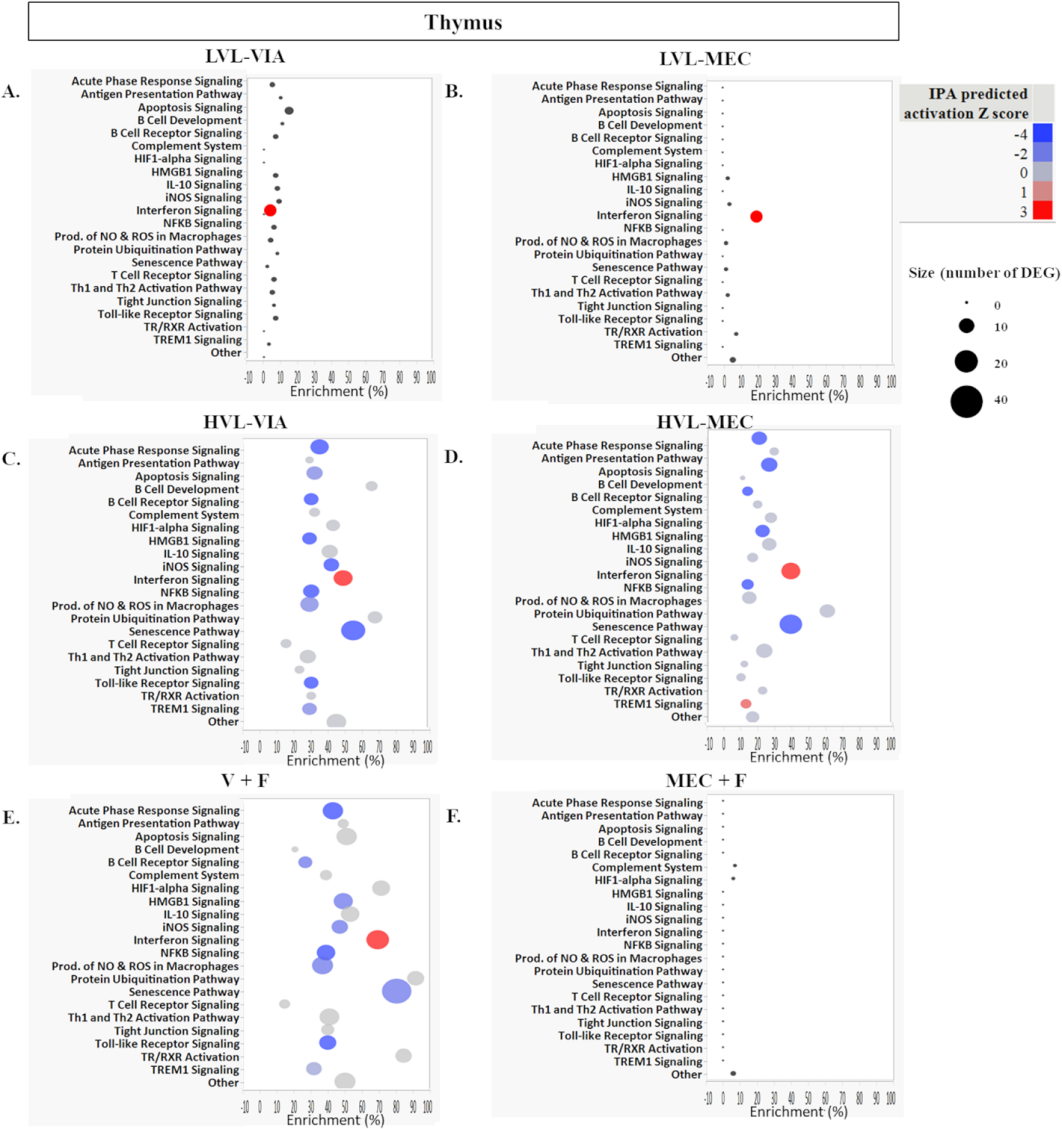
Pathway enrichment of differentially expressed genes in the fetal thymus. A) low viral load with viable (LVL-VIA) contrasted to control (CTRL) fetuses. B) LVL with meconium staining (LVL-MEC) contrasted to CTRL fetuses. C) high viral load with viable (HVL-VIA) contrasted to CTRL fetuses. D) HVL with meconium staining (HVL-MEC) contrasted to CTRL fetuses. E) Virus positive fetus aka V + F (LVL-VIA, LVL-MEC, HVL-VIA, and HVL-MEC) contrasted to virus negative fetus V - F [UNINF, placenta only with VIA (PLCO-VIA), and PLCO-MEC]. F) Meconium stained fetus aka MEC + F (PLCO-MEC, LVL-MEC, and HVL-MEC) contrasted to viable fetus aka MEC - F (UNINF, PLCO-VIA, LVL-VIA, and HVL-VIA). The percent enrichment was calculated as the [(number of DEG assigned to a given pathway by IPA + the number of DEG manually assigned)/(total number of genes assayed on the NanoString in the given pathway)*100]. The pathway analysis plotted with pathway name on the y axis, enrichment (%) on the x, bubble size as the total number of DEG in each pathway, and the color as the IPA predicted activation Z score with blue deactivated and red activated

Pathway enrichment and activation scores for the 22 targeted pathways in placenta and thymus are shown in Figs. 6 and 7, respectively. Across both tissues in every group, except for MEC + F, we identified a strong activation of the Interferon Signaling Pathway (Figs. 6 & 7), driven by the core responsive genes presented previously. In the placenta, the LVL-VIA group pathways were minimally impacted except for Interferon Signaling (Fig. 6a). In the placenta, the Antigen Presentation Pathway and Protein Ubiquitination Pathway were most enriched the LVL-MEC group; Acute Phase Signaling, NFKB, and Senescence Pathways were deactivated; B Cell Receptor Signaling, Production of ROS in Macrophages, and TREM1 Signaling were activated (Fig. 6b). In the placenta, we observed similar patterns in the HVL-VIA as in LVL-MEC with the additional activation of Apoptosis Signaling (Fig. 6c). In the placenta, we mostly detected activation associated with Apoptosis, B Cell Receptor Signaling, iNOS, NFKB, Production of NO and ROS in Macrophages, and TREM1 Signaling (Fig. 6d) in the HVL-MEC group. For placenta in the V + F group, one unique result compared to other contrasts was the strong enrichment of 83% in HIF1-alpha signaling (Fig. 6e) that was not found in other contrast groups (i.e., Antigen Presentation and Ubiquitination were strongly enriched in this group and other contrasts). In placenta, the MEC + F group pathways were minimally impacted with only 7 DEG identified but the strongest enrichment of 20% in the Apoptosis Signaling pathway (Fig. 6f). In the thymus, the LVL-VIA and LVL-MEC group pathways were minimally impacted except for Interferon Signaling (Fig. 7a & b). Similar patterns of pathways were observed between the thymus of HVL-VIA and HVL-MEC fetuses with deactivation of Acute Phase Signaling, Apoptosis, B Cell Receptor, HMGB1 Signaling, NFKB Signaling, and Senescence pathway (Fig. 7c & d). In comparison in the thymus, we detected a 70% enrichment in HVL-VIA, and only 10% enrichment in HVL-MEC, in B cell Development, and deactivation in HVL-VIA and activation in HVL-MEC in TREM1 Signaling (Fig. 7c & d). General patterns in the pathways impacted in the thymus include a high percent enrichment in Protein Ubiquitination Pathway and Senescence Pathway; many pathways with predicted Z scores were deactivated (Fig. 7c & d). In the thymus, the V + F group showed similar patterns of enrichment and activation as the HVL groups, but one key difference was the detection of 90% enrichment in Thyroid hormone receptor/retinoic acid receptor (TR/RXR) Activation, which was not detected to the same extent in any one individual contrast to CTRL (Fig. 7e). In the thymus, the MEC + F group pathways were minimally impacted, likely because only 3 DEG were detected in this contrast, with each DEG belonging to a separate pathway in the form of Complement System, HIF1-alpha Signaling, and Other pathway (Fig. 7f).

## Discussion

Our study characterized genes and pathways associated with variation in VL and preservation status in fetuses. Our main findings were: 1) the host immune response was initiated only in fetuses with detectable levels of PRRSV; 2) upon infection of fetal thymus, a set of core responsive IFN-inducible genes (*CXCL10*, *IFIH1*, *IFIT1*, *IFIT3*, *ISG15*, and *MX1*) were strongly upregulated in both tissues. V - F (contrasted with V - F) had numerous pathways deactivated including Acute Phase Response, B Cell Receptor Signaling, HMGB1 Signaling, iNOS Signaling, NFKB Signaling, Production of RO and NOS in macrophages, Senescence Pathway, and TLR Signaling in both tissues; 3) gene expression associated with VL was more accurately assessed in the thymus than placenta; the strong downregulation of genes in the B Cell Development pathway may be a major mechanism of PRRSV dysregulation of host immunity associated with high VL; and 4) gene expression associated with fetal demise was more accurately assessed in the placenta than the thymus; potential biomarkers of susceptibility (*NFKB2*, *NFKBIA*, and *FASLG*) were identified that may contribute to fetal death. These results advance our understanding of the fetal response to PRRSV infection one step further by characterizing tissue specific gene expression patterns associated with host resistance, resilience, and susceptibility. The fetal classifications used in the current study are based on previous work in our group [16]. We classified the UNINF, PLCO-VIA, PLCO-MEC groups as resistant as they have avoided or minimized infection suggesting some capacity to prevent viral entry/replication [17]. The LVL-VIA and HVL-VIA were classified as tolerant/resilient as they remain uncompromised in the face of viral infection [18] whereas the LVL-MEC and HVL-MEC were considered susceptible as they are neither able to limit viral replication and/or survive it [17]. A limitation of our classifications is that we are looking at a snapshot in time at 12 DPI. We cannot be certain what the ultimate outcome (i.e., VL and meconium staining status) of these fetuses would be if the pregnancy went to term. Additionally, the placenta has been infected with PRRSV (except for the UNINF group) for a longer period compared to the thymus due to the nature of the transplacental infection model. Thus, interpretation of the results of our study carefully considered the experimental design.

### Rational for tissue selection and experimental methods

The placenta functions to protect the fetus while providing critical nutrients and oxygen from the dam to the fetus. We chose to investigate the placenta to gain a better understanding of the role it plays in fetal protection and transmission of PRRSV, critical questions that remain unanswered. The thymus primarily functions as the site for T cell progenitor production and is an important site of PRRSV replication in the fetus [19], indicating the importance of this tissue in understanding host response to infection. We used a targeted gene approach assaying 286 genes using NanoString technology, which is a more sensitive measure of gene expression and less dependent on RNA quality as compared to RNAseq. NanoString analyses are highly dependent on the codeset used. They require considerable expertise to assemble immune gene codesets properly and may miss genes that are not expected to be associated with PRRSV infection. Thus, NanoString analyses may be less useful for hypothesis generation when compared to RNAseq. Future RNAseq analyses of these samples, and potentially additional fetal tissues from these individuals, could identify additional genes and pathways involved in this complex of host-pathogen interactions. All 22 pathways we investigated were enriched in one or more contrasts in this study, providing evidence for the value of the selected set of genes and their associated pathways.

### Importance of fetal infection status on the initiation of host response

A major finding of our study was that PRRSV must be detected in the fetus to initiate a host immunologic response in both the placenta and thymus. Our results show that once fetuses are infected, and especially in HVL fetuses, a strong immune response is initiated as reported previously [13, 19]. However, we detected no DEG in virus negative fetuses (i.e., UNINF, PLCO-VIA, and PLCO-MEC each contrasted to CTRL) in either tissue, indicating the regulation of immune-related gene expression, at least at 12 DPI, is not responsible for generating the resistant fetal phenotype. We anticipated low responsiveness in the thymus of virus negative fetuses but not in the placenta because: 1) virus was detected in the placenta in PLCO groups, 2) the MFI plays an important role limiting fetal exposure to PRRSV, and 3) the placenta has innate immune response capabilities and produces cytokines that play an important role in all stages of pregnancy in pigs [20, 21]. The placenta is also rich in trophoblast cells, which cover most of the placental surface and function to recognize pathogens through TLRs, produce cytokines, and recruit immune cells [22]. In contrast to our results, a previous study using RNAseq reported 864 and 121 DEG in the MFI (endometrium plus placenta) and thymus, respectively, in the UNINF vs CTRL contrast using RNA-seq at 21 DPI [11]. The contrasting results between our study and the Wilkinson study may be explained by timepoint (12 DPI vs 21 DPI), gene expression platform (NanoString vs RNAseq), and/or tissues analyzed (placenta vs MFI, the combined endometrium and placenta). The presence of the endometrial tissue, with its own unique transcriptomic signature, may have not only added additional DEGs but also further obscured others due to relative abundance in such a sample. Alternatively, the lack of response of the placenta, while fetuses are still uninfected in the current study, may be caused by PRRSV modulating the host immune response [1, 23]. More research should be done to probe this mechanism. Although our study did not identify genes and their expression patterns that are associated with resistance, we do report those associated with tolerance and susceptibility.

### IFN response and importance in host immunity

Our results show in virus infected fetuses (LVL-VIA, LVL-MEC, HVL-VIA, and HVL-MEC compared to CTRL) a coordinated and strong upregulation of IFN-inducible genes regardless of fetal VL or preservation status in both placenta and thymus, indicating potential crosstalk in immune activation between the two tissues. A hallmark of host response to viral infection, IFNs are cytokines released from virus infected cells that activate host immunity to combat infection. The pig has IFNs of Types I, II, and III, all of which mainly function to induce a cascade of cellular responses that result in transcriptional activation of immune-related genes via STAT and NF-κB. The pig has many duplicated and unique IFN genes, many of which were tested herein (*IFNA*, *IFNB*, *IFND*, *IFNE*, *IFNG*, *IFNK*, and *IFNW*) along with the receptor *IFNGR1*. Previous research has shown that the level of type I IFNA protein in growing pigs is correlated with favorable immune response to PRRSV infection [24]. Additionally, IFNB protein is induced variably in PRRSV infected porcine alveolar macrophages in vitro [25].

While IFNA and IFNB are the most often studied with regards to viral response, many of the other type I IFNs found in swine have been shown to exhibit antiviral properties with regards to PRRSV [26]. It is, however, worth noting that during at least the early stages of pregnancy the porcine conceptus, like other livestock species, uses type I (IFND) and type II (IFNG) IFN along with the canonical Interferon Signaling pathway to communicate with and alter the behavior of the endometrium [27]. The role of such signaling during later gestations remains unclear, however, it stands to reason such signaling pathways may still modulate functionality at the MFI during late gestation. Here we observed two type I IFNs, *IFNW4/5* and *IFNK*, among the top loaded genes in the placenta principal component 2. Component 2 was found to rather clearly differentiate between VIA and MEC fetuses regardless of VL. In the context of this component both genes were upregulated in viable fetuses and downregulated in their MEC counterparts. It is thus possible that expression of these IFNs may offset the observed significant decrease in placental *IFNE* associated with viral infection of the fetus and, thereby, play a vital role in mediating fetal resilience at the placenta. Another study found high levels of gene expression of type I IFNs (*IFNA* and *IFNB*) in PRRSV infected fetuses compared to non-infected fetuses and the expression was poorly correlated with serum type I IFNs at the protein level [13], indicating a post-transcriptional modification is occurring. In our study, we found a non-consistent pattern of IFN expression across fetal groups and tissues, indicating IFNs themselves may not be good predictors of fetal response to infection. In the placenta, *IFNG* was upregulated in the HVL-VIA fetuses while *IFNE* was downregulated in the fetuses of V + F contrast. We also found *IFNB1* to be increased in placenta HVL-MEC, in the thymus HVL-VIA. In the placenta, *IFNG1R* was downregulated in LVL-MEC, HVL-VIA, HVL-MEC, and in V + F contrast.

The activation of the Interferon Signaling Pathway reported herein was associated with the upregulation of core IFN-inducible genes, *CXCL10*, *IFIH1*, *IFIT1*, *IFIT3*, *ISG15*, and *MX1*, in both tissues in every fetal group that is virus positive (LVL-VIA, LVL-MEC, HVL-VIA, and HVL-MEC each compared to CTRL). While we do not know which IFNs may be driving the expression of these IFN-inducible genes, it may be that IFNs produced in the blood or other lymphatic tissues are transported to the placenta and thymus causing increased expression of IFN inducible genes. This dysregulation of host immunity by PRRSV infection, supports previous works in this area [13, 28–30].

### Dysregulation of immune-related pathways

Our data show that the placenta and thymus of V + F (vs V - F) deactivate Acute Phase Response, B Cell Receptor Signaling, HMGB1 Signaling, iNOS Signaling, NFKB Signaling, Production of RO and NOS in Macrophages, Senescence Pathway, and TLR Signaling. Previous work, typically performed in vitro, has shown that PRRSV can induce many of these pathways [31–37]. Our study is a single snapshot in time (12 DPI) and it may be that the dysregulation of these pathways is time dependent. A commonality of several of these pathways identified in the current study is cellular signaling through the JAK-STAT, MAPK/ERK, and PI3K/AKT/mTOR pathways. Previous work has shown that PRRSV inhibits JAK-STAT signaling through dysregulation of cytokines reviewed in [38]. These complex pathways are integrated via JAK proteins that phosphorylate cytokine receptors, activate MAPK, NFKB and PI3K proteins, and transcriptionally regulate themselves or other proteins downstream [39]. Specifically, in both placenta and thymus V + F groups we report a downregulation at the receptor (*TLR4*), cytoplasmic signaling (*JAK2*, *SOCS3*, *SOCS6*, *PIK3CG/PIK3C2B*), and nuclear signaling levels (*MAP2K4*, *MAP3K7*, *MAPK14*, and *NFKB*). These data suggest that PRRSV infected fetuses have multilevel and widespread silencing of transcriptional immune-related regulatory pathways, which may prevent efficient response to virus.

Differences between tissues in the V + F (vs V - F) include the dysregulation of the Antigen Presentation Pathway and the TR/RXR Activation Pathway in the placenta and thymus, respectively. Antigen presenting cells (APCs) play a crucial role in bridging innate and adaptive immunity by pathogen recognition, processing, and stimulation of T cells via major histocompatibility complex (MHC) presentation [40]. Our data show the dysregulation in the placenta of V + F of Antigen Presentation Pathway through upregulation of *B2M*, *PSMB8*, *PSMB9*, *TAP1*, and *TAP2* but downregulation in the receptor *MHCI-related*. The APCs present in the placenta in V + F (vs V - F) may be infected with PRRSV but may not be interacting with T cells efficiently due to low MHCI protein expression and lack of *IFNG* induction. Interestingly, VL in fetal thymus is positively correlated with the number of PRRSV infected CD163+ and CD169+ cells in the MFI but, unexpectedly, the relationship of CD163+ cell counts in placenta was negatively correlated with fetal thymus VL [8]. Our study design investigates gene expression at the tissue level and thus, the changes in gene expression observed herein may be due to changes in numbers of specific cells (e.g., APCs and T cells), transcriptional changes in cells already present, or a combination of both. Regardless, our data show in detail that PRRSV must be replicating in the fetus to initiate changes in the Antigen Presentation Pathway in the placenta. Future research could use single cell gene sequencing to investigate APCs located in the placenta in virus infected fetuses.

In the thymus of V + F (vs V - F) we show a dysregulation of the TR/RXR Activation pathway. Thyroid hormones regulate a wide range of processes such as growth, development, and metabolism; this pathway has recently been shown to be dysregulated during both maternal and fetal response to PRRSV infection. The triiodothyronine (T3) hormone initiates cellular response by binding the nuclear receptors, THRa and THRb, to directly regulate gene expression. Our data show in the thymus of V + F, downregulation of thyroid hormone receptors (*THRa* and *THRb*), along with the critical outer ring deiodinase (*DIO2*) required to convert T4 to the more bioactive T3. In addition, three of the transmembrane transporters known to aid in the traffic of T3 and T4 into the cytoplasm (*SLC16A10*, *SLCO1C1*, *SLC16A2*) were also down regulated in V + F. Interestingly, we found a strong upregulation in the *DIO3*, an inner ring deiodinase which inactivates thyroid hormone by converting T4 to a comparatively inert rT3 and converting highly bioactive T3 to the metabolite T2, in various placenta contrasts but not in the thymus. Because of thyroid hormone’s role in growth, it could be that fetuses infected with PRRSV are allocating more resources to immunity rather than growth. Complicating matters further is the finding that increased fetal intrauterine growth is associated with a more susceptible phenotype to PRRSV infection [41], possibly indicating a delicate balance in resource allocation of fetuses infected with PRRSV. Alternatively, non-intrauterine growth restricted (IUGR) fetuses could begin infection sooner than IUGR fetuses. More research should be done on fetal thyroid hormone dysregulation to better understand the interplay between immunity and growth.

Our unique dataset allowed characterization of the gene expression differences related to fetal VL (LVL-VIA vs HVL-VIA) providing insight into mechanisms of resilience and/or susceptibility. We predicted that HVL fetuses would have a poorer outcome compared to LVL based on previous studies [10], indicating that fetal compromise and death is strongly related to VL. Despite virus detected in fetuses within the LVL-VIA, Interferon Signaling is the only pathway impacted in both the placenta and thymus. It could be that an immune response at an earlier timepoint limited the VL in these fetuses. Alternatively, delayed infection is a viable alternative, in that the LVL fetuses could have been infected a few days later than the HVL fetuses and the virus had not had enough time to replicate. By comparison, in the HVL-VIA we observe a strong downregulation of numerous immune pathways (vs CTRL), especially in the thymus. In the HVL-VIA group we did not detect as clear of a picture of host dysregulation in placenta as in the thymus.

### Assessment of fetal demise and viral levels by gene expression

The downregulation of pathways and genes gives insight into ways the fetal host fails to limit viral replication. Dysregulation of B Cell Receptor Signaling, NFKB Signaling, iNOS Signaling, and HMGB1 Signaling in the thymus may contribute to increases in VL. We found a large number (*N* = 58) of DEG identified exclusively in the thymus of HVL-VIA fetuses (vs LVL-VIA, LVL-MEC, and HVL-MEC fetuses), all of which were downregulated compared to CTRL. Among these downregulated genes were *CD19*, *CD25B*, *CD55*, *CD79A*, *CD79B*, and *CD83*. The cluster differentiation (CD) genes have pleotropic immunologic roles but are well known as receptors often used to differentiate immune cell subtypes. The pathway analysis for this group of fetuses showed a 70% enrichment in B Cell Development that was largely unimpacted in any other fetal group. Our study investigated fetal response at 12 DPI and therefore, the fetuses could have been exposed to replicating PRRSV for 5 days already [16]. These genes may be considered markers of the inability to limit viral replication which could be associated with invasion of the thymus by immune cell populations or maturation of resident immune cells.

Finally, a major finding of our study was that gene expression profiles in the placenta more accurately assessed fetal demise compared to gene expression patterns in the thymus. Although we detected no DEG in either tissue in the comparison of PLCO-MEC versus CTRL, we observed a clear separation of the PLCO-MEC fetal groups across component 2 in both tissues. This fetal grouping is classified in the resistant category for the ability to limit/prevent viral infection. However, fetuses are clearly compromised via meconium staining for which the cause is not completely understood. The clear separation across component 2 may be indicative of conserved gene expression patterns consistent with fetal demise, regardless of fetal viral load. Although PLCO-MEC represents a relatively small proportion (i.e., in the current study *N* = 4 and *N* = 5 for the placenta and thymus, respectively) of the overall array of fetal outcomes, exploring their response further may be warranted. Our results clearly show the placenta has more unique changes in pathways between VIA and MEC fetuses compared to the thymus. Interestingly, we detected a robust gene expression response in the placenta LVL-MEC group, while the thymus remained relatively silent in both LVL-VIA and LVL-MEC groups. Our data revealed the clear separation of MEC from VIA fetuses on component 2 of the PCA for the placenta but not for the thymus. The top negative loading genes in the placenta for this component were *IFNW4/W5*, *IFNK*, *MBL1*, *MASP1*, and *IL12B* (Supplemental Table 3). The *IFNW4/5* and *IFNK* were discussed above. The MBL1 protein may play a role in Complement activation [42]. MASP1 and IL12B proteins function in the Complement System and have pleotropic functions (HMGB1, Th1 and Th2 Activation, and TLR Signaling), respectively. Interestingly, all these genes were more lowly expressed in MEC fetuses compared to VIA fetuses. The direction of expression could be interpreted in two ways; reduced gene expression contributes to a susceptible phenotype (MEC) or higher gene expression contributes to a resistant/resilient phenotype (VIA). It could be that higher gene expression, especially that of the IFNs, may have a protective effect in the placenta. However, none of these genes were independently identified as DEG in any comparison groups indicating more research should be done on these biomarkers. In addition, in the differential expression analysis of MEC + F we identified 7 and 3 DEG in the placenta and thymus, respectively. Of these 7 DEG in the placenta, we found *NFKB2*, *NFKBIA*, and *FASLG* to have consistently higher expression and statistically significant patterns in fetuses with meconium staining (PLCO-MEC, LVL-MEC, HVL-MEC) compared to viable (UNINF, PLCO-VIA, LVL-VIA, and HVL-VIA) fetuses. The NFKB2 and NFKBIA proteins are highly pleotropic but are best known for their involvement in the NFKB Pathway, a well-characterized pathway initiated during host infection [43]. Interestingly, all three of these genes are in the Apoptosis Pathway. Across all groups of virus positive fetuses in the placenta, we see a strong activation and enrichment of genes in the Apoptosis and Ubiquitination Pathways. PRRSV causes apoptosis in the maternal uterine epithelium and fetal trophoblast epithelium as well as surrounding cells in late gestation [6, 44].

The question of PRRSV infection in the fetus as the cause of demise has been previously questioned based on the understanding that fetal lesions are infrequently observed [19]. However, it is now understood that the fetus responds physiologically and immunologically to PRRSV infection in the absence of pathology [11, 13, 14, 45]. Previous reports show a positive association between fetal survival during PRRSV infection and reduced intrauterine growth [16], of which increased apoptosis and increased cellular senescence could contribute to reduced fetal growth. Interestingly, we report changes in gene expression that downregulate genes in the Cellular Senescence Pathway in the thymus while Apoptosis Pathway in the placenta were activated. Cellular senescence is the reduction of cell division through various cell cycle checkpoints and is typically activated by decreased telomere length, or cellular stress, while it is sometimes decreased in cancerous cells via mTOR regulatory mechanism. Our results show that very different cellular survival/proliferation mechanisms are possibly occurring between the two tissues, with high cell death in the placenta and high cell proliferation in the thymus, giving further evidence to support that gene expression in the placenta more accurately assesses fetal demise while gene expression in the thymus more accurately assesses VL. However, the temporal aspect of our study must be considered because the placenta is infected with virus for a longer period in transplacental challenge compared to the thymus. For the first time, we report *NFKB2*, *NFKBIA*, and *FASLG* as potential biomarkers in the placenta that may contribute to fetal demise (MEC). Taken together, our study provides unprecedented insight into fetal response to PRRSV infection with a complex interplay between placenta and thymus.

## Conclusions

It is uncertain why fetuses, within a single PRRSV infected gilt, have such large variations in VL and preservation status. Our study probed the molecular mechanisms behind these differences. We found that the fetal immune response was initiated in the fetus and at the MFI, specifically in the placenta, only after virus was at detectible levels in the fetus, indicating a complex process of host-pathogen interaction. Significant crosstalk between fetal tissues and the placenta was occurring during fetal infection as several genes and pathways were impacted similarly. We found gene expression in the thymus more accurately assessed fetal VL than the placenta. Moreover, infected fetuses unable to reduce VL (i.e., the HVL groups) showed a strong downregulation of genes in numerous immune pathways. Gene expression in the placenta more accurately assessed fetal demise than the thymus. The biomarkers identified here may be used as a first step to breeding pigs for improved animal health and/or to development of anti-viral therapeutics.

## Methods

### Animals, experimental challenge with PRRSV, and fetal groupings

The aim of our study was to probe the molecular mechanisms of disease resistance, tolerance, and/or susceptibility of fetuses to PRRSV. Purebred Landrace gilts were purchased from a high-health nucleus heard (Fast Genetics Inc., Spiritwood, SK) and were artificially inseminated with homospermic semen from Yorkshire boars (Fast Genetics Inc., Spiritwood, SK) as described previously in great detail [46]. Authors were aware of group allocation at the different stages of the experiment. Pregnant gilts at 84 (± 0.4) days gestation were randomly chosen to be infected (*N* = 31) with PRRSV2 (NVSL97–7895) or mock infected (*N* = 7) as described previously in detail [10]. At 12 DPI, gilts were euthanized, and fetal preservation status determined. Humane euthanasia of animals was completed as follows. We used a solution of 30 mL of pentobarbital sodium (16,2000 mg) per gilt diluted with equal parts sterile water. This solution was injected intravenous into the vena cava vein (50%) and when deeply sedated (i.e., in lateral recumbency), the remaining solution was administered via intra cardiac injection. This provides ~ 80 mg/kg, sufficient to euthanize the gilt as well as fetuses as pentobarbital crosses the placenta quickly. This was followed by cranial captive bolt shot and exsanguination. MFI samples were carefully dissected so that placenta VL could be independently assessed along with thymus and serum as described previously [16]. Fetuses were categorized by preservation status and PRRS VL (Fig. 1, Table 1) into disease resistance, tolerance, and susceptibility groups: CTRL, UNINF, PLCO-VIA, PLCO-MEC, LVL-VIA, LVL-MEC, HVL-VIA, or HVL-MEC. Fetuses that were not alive at the time of sampling were excluded from the dataset. A total of 92 placenta and 94 thymus samples were collected and assayed for gene expression. While the number of samples per group was not balanced and ranged from 4 to 16 individuals per group (Table 1) it was reflective of the fetal population.

### RNA isolation, quality control, and gene expression analysis

RNA was isolated from placenta and thymus tissues using the RNeasy mini kit (P/N 74106, Qiagen). Isolated RNAs were checked for quality and quantity using an Agilent 2200 TapeStation (Agilent Technologies). The RNA integrity number (RIN) values were on average 5 in the placenta and 8 in the thymus. A total of 286 test genes and 10 housekeeping genes were chosen for gene expression quantification on the NanoString nCounter array (NanoString Technologies) using custom-made probes. The test genes were chosen based on 22 IPA-verified immune pathways previously shown or hypothesized to be altered by PRRSV infection (Table 3). Samples were run on the NanoString using manufacturer’s instructions with the nCounter Master kit. Samples were randomized on the NanoString chips in a block design where approximately the same number of fetal samples from each group were run on each chip. The nCounter analysis system produces discrete count data for each gene assayed within each sample. We used the NanoString software nSolver Analysis Software (version 3.0, NanoString Technologies), following manufacturer’s instructions. The nSolver corrects for background based on negative control samples, performs within sample normalization based on positive control probes, and performs normalization across samples using the median expression values of housekeeping genes. In the current study the normalization across samples was based on inclusion of 10 housekeeping genes (i.e., *GAPDH*, *HMBS*, *HPRT1*, *IPO8*, *MAU2*, *RPL32*, *RPL4*, *SDHA*, *STX5*, and *TOP2B*) providing confidence in our normalization method, addressing a potential concern of others [47]. Novel statistical methods have been produced to perform differential expression analysis on normalized nCounter data assuming a generalized linear model [48, 49]. To determine the most appropriate statistical software to analyze the normalized data in the current study we took a statistical approach. The normalized nCounter data was screened for each gene, within fetal group, within tissue and tested for a Normal Distribution using the Shapiro-Wilks statistical test in JMP Software where the Ho (null hypothesis) is that the data follows a Normal distribution, and thus, *P* < 0.05 reject the null hypothesis. Based on this approach, we determined the data largely followed a Normal distribution. Thus, we chose to use Limma software which assumes a Normal distribution and uses linear models to calculate DEG using all normalize count data within each tissue. Normalized counts in the placenta and thymus for all genes are found Supplemental Tables 5 and 6, respectively. All samples passed internal NanoString QC. A total of 10 samples (6 from the placenta and 4 from the thymus) were removed because they were determined to be extreme outliers characterized by extremely high normalized counts compared to all other samples in nearly every gene as well as extremely low raw counts in the housekeeping genes.

### Differential gene expression analysis

Normalized count data for the 286 test genes was used as input into the Limma Zoom R package [50] with the following gene expression model Y_norm counts_ = Group + Dam(random) + Error. The count data were checked for distribution (as stated above) as Limma software assumes the data are in a Normal distribution. The experimental unit was an individual tissue sample from a single fetus. Analyses were run separately within each tissue and all data for that tissue was used during each contrast. First, each group was contrasted with the CTRL group i.e., UNINF vs CTRL, PLCO-VIA vs CTRL, PLCO-MEC vs CTRL, LVL-VIA vs CTRL, LVL-MEC vs CTRL, HVL-VIA vs CTRL, and HVL-MEC vs CTRL. Additionally, virus positive fetus’s aka V + F were contrasted with V - F (LVL-VIA + LVL-MEC + HVL-VIA + HVL-MEC vs UNINF + PLCO-VIA + PLCO-MEC). Similarly, meconium stained fetus’s aka MEC + F were contrasted with MEC - F (PLCO-MEC + LVL-MEC + HVL-MEC vs UNINF + PLCO-VIA + LVL-VIA + HVL-VIA). Multiple testing was corrected using Benjamini & Yekutieli [51] correction with adjusted *P* value ≤ 0.05 considered significant. No log2FC cutoff was used.

### Visualization of DEG

Proportional-area Venn diagrams based on the Euler method [52] were generated to identify shared and unique DEG between various contrast groups using JMP Pro 15.0.0 (SAS Institute). PCA was used to understand the clustering relationship between fetal groups within tissue using JMP software. Using the individual groups vs CTRL (i.e., UNINF, PLCO-VIA, PLCO-MEC, LVL-VIA, LVL-MEC, HVL-VIA, and HVL-MEC) log2FC results from all 286 genes within tissue was used as input into the PCA with default parameters. The top positively and negatively loaded genes placed onto component 1 and 2 within tissue were plotted to investigate informative expression patterns across fetal groups to reveal factors contributing to the separation of the principle components.

Heatmaps were generated to visualize and identify informative expression patterns using hierarchical clustering with default parameters in JMP Pro 15.0.0 (SAS Institute). Input data was based on the unique and shared pathways in the placenta and thymus in the V + F based on the Venn Diagram result in Fig. 2c. Data on the log2FC of all 286 genes for every contrast group to CTRL were clustered.

### Pathway analysis

IPA Core Analysis function was used to (accessed February 27, 2020) investigate the impact of our DEG on the 22 targeted pathways as well as predict if pathways had positive Activation Z scores (activated), negative Activation Z scores (deactivated), or were not changed. The activation Z scores are predictions based on the directionality of DEG described previously [53]. Additionally, the percent enrichment was calculated as the [(number of DEG assigned to a given pathway by IPA + the number of DEG manually assigned)/(total number of genes assayed on the NanoString in the given pathway)*100]. The pathway analysis was plotted with pathway name on the y axis, enrichment (%) on the x, bubble size as the total number of DEG in each pathway, and the color as the IPA predicted activation Z score.

## Supplementary information

**Additional file 1 **: **Figure S1.** Diagram illustrating how the fetal groupings were assigned.

**Additional file 2 **: **Figure S2.** Venn diagrams of DEG within group between tissues. Sizes based on total numbers of DEG for a given contrast. Placenta is on the left and thymus is on the right. A) Red is the LVL-VIA. B) Yellow is LVL-MEC. C) Blue is HVL-VIA. D) Green is HVL-MEC.

**Additional file 3 **: **Table S1.** Gene names. A total of 286 test genes and 10 housekeeping genes were assayed for mRNA expression levels on the NanoString platform. **Table S2.** Differential expression analysis results for all genes and all contrasts. The results are organized by gene then by contrast group within tissue. The included columns are log2FC: the log2 fold change value in the test group compared to the contrast group, AveExpr: the average count expression value for the gene across all samples, t: the T statistic for a given gene and contrast, *P* Value: the calculated *P* value for a given gene and contrast, adj.P.Val: the P value adjusted for multiple testing using the Benjamini Yekutieli correction method, and B: The B statistic for the log odds that the gene is differentially expressed. **Table S3.** Principle component loading gene values for placenta and thymus. **Table S4.** The Activation Z scores for all recognized pathways in the IPA software. The results are organized by IPA pathway, then by contrast group within tissue. The core analysis was done on individual contrast groups using DEG as input. **Table S5.** Placenta normalized counts by gene. **Table S6.** Fetal thymus normalized counts by gene.

## Data Availability

All datasets used for this study are included in the supplementary materials.

## Abbreviations

APC: Antigen presenting cells
CD: Cluster differentiation
CTRL: Mock infected control
DEG: Differentially expressed genes
DPI: Days post infection
HVL: High viral load
IFN: Interferon
IPA: Ingenuity pathway analysis
IUGR: Intrauterine growth restriction
LVL: Low viral load
MEC: Meconium stained fetus
MEC - F: Viable fetus
MEC + F: Meconium stained fetus
MFI: Maternal fetal interface
MHC: Major histocompatibility complex
PCA: Principle component analysis
PLCO: Placenta only
PRRS: Porcine reproductive and respiratory syndrome
PRRSV: Porcine reproductive and respiratory syndrome virus
PRRSV2: Porcine reproductive and respiratory syndrome virus type 2
R: Resilient
RIN: RNA integrity number
S: Susceptible
SER: Fetal serum
T: Tolerant
T3: Triiodothyronine
TR/RXR: Thyroid hormone receptor/retinoic acid receptor
UNINF: Uninfected
V - F: Virus negative fetus
V + F: Virus positive fetus
VIA: Viable fetus
VL: Viral load
