# Supplementary figures and images for: Differential responses in placenta and fetal thymus at 12 days post infection elucidate mechanisms of viral level and fetal compromise following PRRSV2 infection
Source: BMC Genomics. 2020 Nov 4;21:763. doi: 10.1186/s12864-020-07154-0 (PMC7640517; doi:10.1186/s12864-020-07154-0)

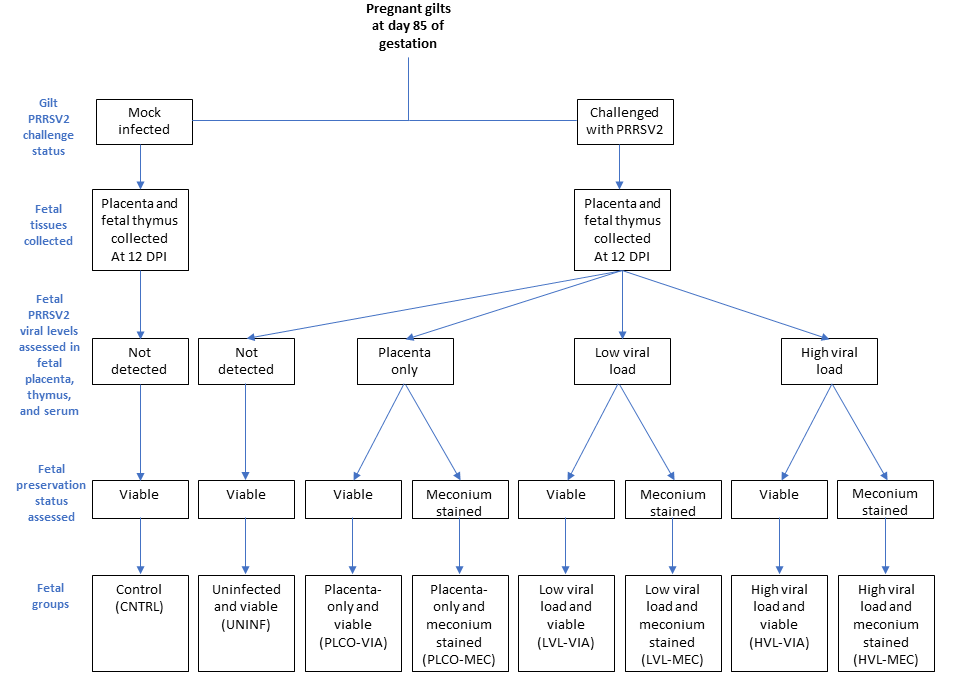

Supplement: Supplementary file 1 — Additional file 1 : Figure S1. Diagram illustrating how the fetal groupings were assigned. [file 12864_2020_7154_MOESM1_ESM.tif]

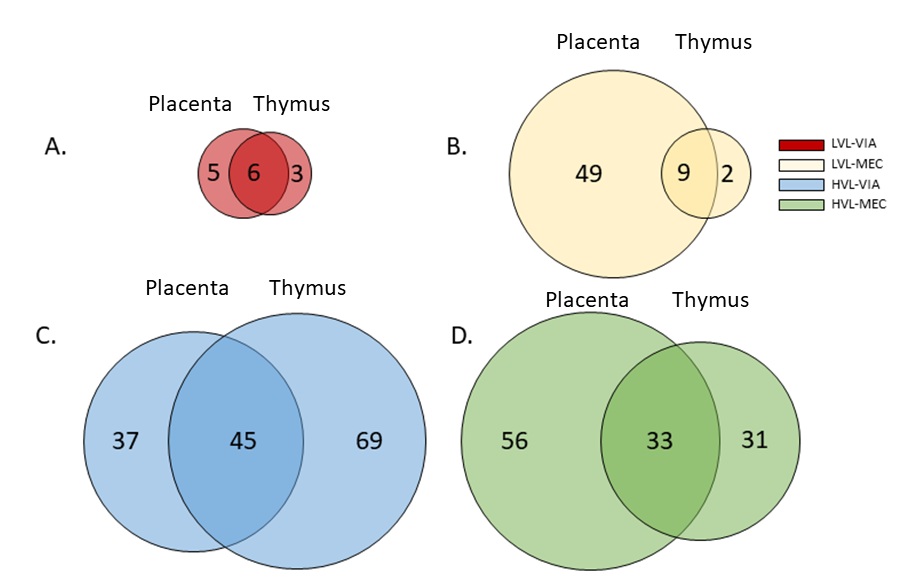

Supplement: Supplementary file 2 — Additional file 2 : Figure S2. Venn diagrams of DEG within group between tissues. Sizes based on total numbers of DEG for a given contrast. Placenta is on the left and thymus is on the right. A) Red is the LVL-VIA. B) Yellow is LVL-MEC. C) Blue is HVL-VIA. D) Green is HVL-MEC. [file 12864_2020_7154_MOESM2_ESM.tif]
